# Declining utilization of urodynamic studies in urological care in Germany: time to say goodbye?

**DOI:** 10.1007/s00345-024-05154-3

**Published:** 2024-07-24

**Authors:** Martin Baunacke, Livia Kontschak, Viktoria Menzel, Markus Grabbert, Angelika Borkowetz, Sherif Mehralivand, Nicole Eisenmenger, Johannes Huber, Christian Thomas, Daniela Schultz-Lampel

**Affiliations:** 1 Department of Urology, TU Dresden, Fetscherstr. 74, 01307 Dresden, Germany; 2 Department of Urology, University of Freiburg, 79106 Freiburg, Germany; 3 Reimbursement Institute, Hürth, Germany; 4 Department of Urology, Philipps-University Marburg, 35043 Marburg, Germany; 5 Southwest Continence Center, Schwarzwald-Baar-Klinikum, 78052 Villingen-Schwenningen, Germany

**Keywords:** Urodynamic studies, Health services research, Continence, Epidemiology

## Abstract

**Introduction:**

The number of urodynamic studies (UDS) has been declining steadily in recent decades, yet the reasons behind this trend remain poorly understood. This study aims to investigate the structural aspects of UDS in urology and explore the factors contributing to this decline.

**Material & methods:**

We surveyed all urological departments performing UDS as well as a representative sample of private practices in Germany in 2023. We examined structural situation, waiting times, capacities and limitations of UDS. All invasive urodynamic examinations were defined as UDS.

**Results:**

In 2019, 259/474 (55%) urological departments in Germany performed UDS. 206/259 (80%) urological departments responded to the survey. 163/200 (82%) urological departments stated that their capacities were exhausted, a main reason being lack of medical and nursing staff. 54.8% urological departments performed more than 50% of their UDS for referring physicians. Urological departments with a low number of UDS/year (≤ 100) showed a shorter waiting time (up to 4 weeks: 49% vs. 30%; *p* = 0.01), reduced UDS capacities (55% vs. 12%; *p* < 0.001) and these capacities were often not fully utilized (25% vs. 9%; *p* = 0.007). 122/280 (44%) office urologists responded to the survey. 18/122 (15%) office urologists performed UDS. Main reasons for not offering UDS were lack of personnel and low reimbursement.

**Conclusion:**

In German urological departments, UDS capacities are consistently fully utilized, primarily due to staffing shortages. This trend towards centralization prompts questions about the role of UDS in urologists’ training.

**Supplementary Information:**

The online version contains supplementary material available at 10.1007/s00345-024-05154-3.

## Introduction

Urodynamic studies (UDS) enable the direct assessment of micturition process and play an important role in the diagnosis of functional disorders of the lower urinary tract [1, 2]. As one of the most intricate examinations in urology, UDS necessitates experience and standardized procedures for reliable results [3, 4]. Previous international studies have indicated a declining trend in UDS utilization. There are few older international studies analysing UDS utilization [5–8]. Our study group analysed UDS utilization in Germany from 2013 to 2019 revealing a notable decrease, particularly in urological departments (-45%) over six years [9]. This prompts inquiry into the underlying cause. On the one hand, studies in last ten to twelve years have questioned the necessity of UDS in certain cases. The ValUE (Value of Urodynamic Evaluation) and VUSIS 2 (the Value of Urodynamics before Stress Incontinence Surgery) trials showed no added value of UDS in the diagnosis of uncomplicated stress urinary incontinence [10, 11]. This results in guideline changes for urinary incontinence made in 2013. The recommendation to “perform UDS prior to surgery for urinary incontinence if there are either symptoms of overactive bladder, a history of previous surgery, or a suspicion of voiding difficulty” was removed [12]. Additionally, findings from the UPSTREAM Trial (Urodynamics for Prostate Surgery Trial: Randomized Evaluation of Assessment Methods) showed no benefit of UDS for men with LUTS in routine diagnostic [13]. Moreover, the resource-intensive nature of UDS, involving time, personnel, and material costs, poses financial challenges as reimbursement often falls short [14].

Hence, the question arises as to whether the decline in urodynamics is attributed to fewer indications or diminished structural provision [14–16]. The aim of this study is to analyse status of urodynamics within the context of urological care in both hospital and outpatient settings in Germany.

## Material & methods

For this study we surveyed urological departments and office urologists. Urological departments that performed UDS in 2019 received a questionnaire. To identify these departments, we used German hospitals’ quality reports by using OPS (Operationen-und Prozedurenschlussel) codes 1-334, including their subcodes. We utilized the analysis tool “reimbursement.INFO” (Reimbursement Institute, Hurth, Germany) to extract data on hospital UDS utilization. To survey office urologists, we compiled a list of all urologists in private practice according to the online information provided by the 17 associations of statutory health insurance physicians (Kassenärztliche Vereinigungen) in Germany. At the time the list was compiled, 3232 urologists were listed (November 2022). This list contains information on the private practice (Medizinisches Versorgungszentrum = health care center, self-employed urologists), federal state and city size. We used this data to create a representative sample with regard to the distribution of the federal state, the population and practice type (*n* = 280).

Urological departments received a questionnaire in May 2023 to evaluate the frequency of urodynamic studies conducted annually (Supp. 1). Subsequently, office urologists received a questionnaire in October 2023 (Supp. 2). Non-responders were contacted a second time, two months later. The survey aimed to gather data on structural situation, waiting times and capacities related to UDS. Furthermore, we analyzed barriers hindering the implementation of UDS. These are medical and nursing staff, structure (infrastructure/space) and remuneration. The term “UDS capacities” in this study is defined about the possibility of using a UDS, which can be limited by various factors.

Due to the nature of the data (publicly accessible hospital quality reports and survey of institutions), ethical approval was not needed. Statistical analysis was performed using t-tests and chi-square tests, with a significance level set at *p* < 0.05. All calculations were performed with “IBM SPSS Statistics 28” (Armonk, NY, USA).

## Results

### Urological departments

**Fig. 1:**
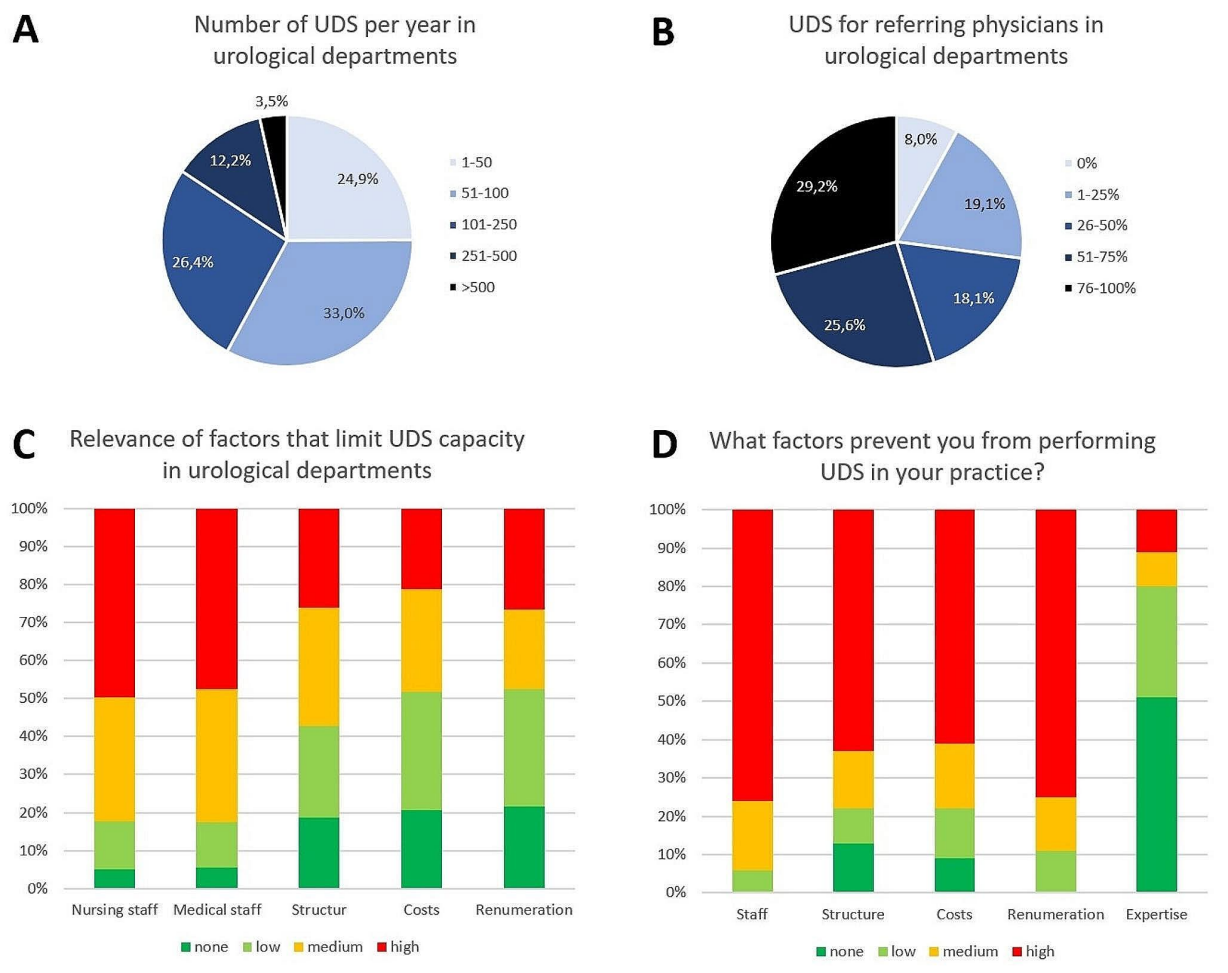
(**A**) number of UDS per year in urological departments (*n* = 197); (**B**) share of UDS for referring physicians in urological departments (*n* = 199); (**C**) Relevance of factors that limit UDS capacity in urological departments (*n* = 151); (**D**) Factors that prevent office urologists to perform UDS (*n* = 97)

In 2019, 259/474 (55%) urological departments in Germany performed UDS. Of these 259 urological departments 206 (80%) responded to the survey. Among the surveyed urological departments, 39/205 (19%) were affiliated with a university hospital and 66/204 (32%) were recognized as certified pelvic floor centres. 25% of all urological departments performed 1–50 UDS/year, 33% performed 51–100 UDS/year, 26% performed 101–250 UDS/year, 12% performed 251–500 UDS/year and only 4% performed more than 500 UDS/year (Fig. 1A). 54.8% of urological departments performed more than 50% of their UDS for referring physicians (Fig. 1B).

163/200 (82%) urological departments state that their capacities were exhausted (Table 1). Figure 1 C illustrates the importance of factors limiting capacity within these urological departments.

**Table 1:** Collective of urological departments performing UDS (*n* = 206)

		Total (*n* = 206)
University hospital (*n* = 205)	Yes	39 (19%)
	No	166 (81%)
Number of beds (*n* = 205)	1–10	5 (2%)
	11–30	71 (35%)
	31–50	95 (46%)
	> 50	35 (17%)
Certified pelvic floor centres (*n* = 204)	Yes	66 (32%)
	No	138 (67%)
Number of UDS/year (*n* = 197)	1–50	49 (25%)
	51–100	65 (33%)
	101–250	52 (26%)
	251–500	24 (12%)
	> 500	7 (4%)
Waiting time for UDS (*n* = 198)	< 1 week	4 (2%)
	1–4 weeks	75 (38%)
	1–3 months	104 (53%)
	4–6 months	10 (5%)
	> 6 months	5 (3%)
Development of the UDS number in the last 5 years (*n* = 199)	Decreasing	73 (36%)
	No change	63 (32%)
	Increasing	63 (32%)
Planning the UDS number over the next 5 years (*n* = 205)	Reduction	16 (8%)
	No change	142 (69%)
	Increase	47 (23%)
Share of referring urologists (*n* = 199)	None	16 (8%)
	1–25%	38 (19%)
	26–50%	36 (18%)
	51–75%	51 (26%)
	76–100%	58 (29%)
Are UDS capacities exhausted? (*n* = 200)	Yes	163 (82%)
	No	37 (18%)
Relevance of factors that limit capacity (0 (none) – 3 (high))	Nursing staff (*n* = 159)	2.3 ± 0.9 2 (0–3)
	Medical staff (*n* = 160)	2.2 ± 0.9 2 (0–3)
	Structure (*n* = 161)	1.7 ± 1.1 2 (0–3)
	Cost (*n* = 160)	1.5 ± 1.0 1 (0–3)
	Renumeration (*n* = 158)	1.5 ± 1.1 1 (0–3)

Urological departments with a low number of UDS/year (≤ 100) were smaller departments (1–30 beds: 49% vs. 18%; *p* < 0.001) and were less often certified as pelvic floor centres (25% vs. 39%; *p* = 0.04). In these low volume departments, waiting times for UDS were shorter (up to 4 weeks: 49% vs. 30%; *p* = 0.01), UDS capacities were reduced (55% vs. 12%; *p* < 0.001) and often not fully utilized (25% vs. 9%; *p* = 0.007). In low volume urological departments relevance of costs and renumeration for limiting UDS were significantly higher than compared to high volume departments (costs: 1.7 ± 1.2 vs. 1.2 ± 1.1, *p* = 0.005, renumeration: 1.7 ± 1.1 vs. 1.2 ± 1.1, *p* = 0.001) (Table 2).

**Table 2:** Comparing urological departments with low and high UDS case numbers (*n* = 190)

		Total (*n* = 190)	Low case number (≤ 100 UDS/a) (*n* = 114)	High case number (> 100 UDS/a) (*n* = 76)	*p* value
Number of beds of the urological department	1–10	5 (3%)	5 (4%)	0 (0%)	**< 0.001**
	11–30	65 (34%)	51 (45%)	14 (18%)	
	31–50	89 (47%)	46 (40%)	43 (57%)	
	> 50	31 (16%)	12 (11%)	19 (25%)	
Certified continence centre (*n* = 188)	Yes	57 (30%)	28 (25%)	29 (39%)	**0.04**
	No	131 (70%)	85 (75%)	46 (61%)	
University hospital (*n* = 189)	Yes	36 (19%)	15 (13%)	21 (28%)	**0.01**
	No	153 (81%)	98 (87%)	55 (72%)	
Waiting time for UDS (*n* = 188)	< 1 week	4 (2%)	3 (3%)	1 (1%)	**0.01**
	1–4 weeks	74 (40%)	52 (46%)	22 (29%)	
	1–3 months	96 (51%)	50 (44%)	46 (61%)	
	4–6 months	10 (5%)	3 (3%)	7 (9%)	
	> 6 months	4 (2%)	4 (4%)	0 (0%)	
Development of the UDS number in the last 5 years (*n* = 189)	Decreasing	72 (38%)	63 (55%)	9 (12%)	**< 0.001**
	No change	59 (31%)	27 (24%)	32 (43%)	
	Increasing	58 (31%)	24 (21%)	34 (45%)	
Are UDS capacities exhausted? (*n* = 189)	Yes	154 (81%)	85 (75%)	69 (91%)	**0.007**
	No	35 (19%)	28 (25%)	7 (9%)	
Planning the UDS number over the next 5 years	Reduction	15 (8%)	11 (10%)	4 (5%)	0.5
	No change	133 (70%)	78 (68%)	55 (72%)	
	Increase	42 (22%)	25 (22%)	17 (22%)	
Share of referring physicians (*n* = 189)	None	15 (8%)	10 (9%)	5 (7%)	0.2
	1–25%	37 (20%)	22 (19%)	15 (20%)	
	26–50%	32 (17%)	22 (19%)	10 (13%)	
	51–75%	48 (25%)	32 (29%)	16 (21%)	
	76–100%	57 (30%)	27 (24%)	30 (39%)	
Relevance of factors that limit capacity (0 (none) – 3 (high))	Nursing staff (*n* = 152)	2.3 ± 0.9 2.5 (0–3)	2.3 ± 0.8 2.0 (0–3)	2.2 ± 1.0 3.0 (0–3)	0.6
	Medical staff (*n* = 153)	2.2 ± 0.9 2.0 (0–3)	2.3 ± 0.8 3.0 (0–3)	2.1 ± 0.9 2.0 (0–3)	0.2
	Structure (*n* = 153)	1.6 ± 1.1 2.0 (0–3)	1.6 ± 1.0 2.0 (0–3)	1.7 ± 1.1 2.0 (0–3)	0.4
	Costs (*n* = 153)	1.5 ± 1.0 1.0 (0–3)	1.7 ± 1.0 2.0 (0–3)	1.2 ± 1.1 1.0 (0–3)	**0.005**
	Renumeration (*n* = 151)	1.5 ± 1.1 1.0 (0–3)	1.7 ± 1.1 2.0 (0–3)	1.2 ± 1.1 1.0 (0–3)	**0.002**

### Office urologists

122/280 (44%) office urologists responded to the survey. Among these, 18 (15%) performed UDS, while 10 out of 122 (8%) possessed a urodynamic device but were not utilizing it. Comparative analysis between office urologists with and without UDS revealed no discernible disparities in either medical office classification (*p* = 0.9) or patient volume per quarter (*p* = 0.4). Office urologists equipped with UDS provide more indications for UDS annually compared to these offices without UDS (> 25 UDS/year: 67% vs. 33%; *p* = 0.01). The difference regarding waiting time didn’t reach statistical significance (weeks: 8.1 ± 5.4 vs. 11.3 ± 7.1; *p* = 0.052) (Table 3).

**Table 3:** Sample size of office urologists with and without UDS (*n* = 122)

		Total (*n* = 122)	No UDS (*n* = 104)	UDS (*n* = 18)	*p* value
Type of private practice (*n* = 119)	Single practice	38 (32%)	33 (33%)	5 (28%)	0.9
	Group practice	25 (21%)	21 (21%)	4 (22%)	
	Community health center	56 (47%)	47 (46%)	9 (50%)	
Patients per quarter (*n* = 121)	< 500	5 (4%)	5 (5%)	0 (0%)	0.4
	500–1000	21 (17%)	17 (17%)	4 (22%)	
	1001–2000	68 (56%)	60 (58%)	8 (45%)	
	> 2000	27 (23%)	21 (20%)	6 (33%)	
Performing UDS	No	94 (77%)	94 (90%)	0 (0%)	**< 0.001**
	Yes	18 (15%)	0 (0%)	18 (100%)	
	No, but I have a UDS device	10 (8%)	10 (10%)	0 (0%)	
Would you perform more UDS with more capacity? (*n* = 118)	Yes	66 (56%)	58 (58%)	8 (44%)	0.3
	No	52 (44%)	42 (42%)	10 (56%)	
Number of UDS/year (*n* = 115)	0–25	71 (62%)	65 (67%)	6 (33%)	**0.01**
	26–50	33 (29%)	24 (25%)	9 (50%)	
	51–100	5 (4%)	5 (5%)	0 (0%)	
	> 100	6 (5%)	3 (3%)	3 (17%)	
Waiting time for UDS (weeks) (*n* = 110)		10.8 ± 6.9 10 (2–30)	11.3 ± 7.1 10 (2–30)	8,1 ± 5,4 8 (4–26)	0.052

Figure 1D highlights the significance of factors influencing the decision of offices to forego UDS. 87/102 (85%) offices opted for UDS to be conducted by urological departments, 14% (14/99) send them to gynaecological departments and 8 out of 98 (8%) to other office urologists.

12/18 (67%) offices with UDS stated that the number of UDS had decreased in the last 5 years, while 28% (5/18) indicated no change and 1/18 (6%) reported an increase of UDS. 11/18 (61%) offices mentioned that their UDS capacities were exhausted.

## Discussion

In 2019, only 55% of German urological departments performed UDS. The primary factors contributing to the limitation of UDS capacities in clinics were identified as staffing constraints, both in nursing and medical personnel. Notably, urological departments with a lower annual count of UDS tended to further reduce their numbers, driven by decreased capacity utilization and shorter waiting times. A relevant part of UDS in hospitals were carried out as commissioned services for external referring urologists. Conversely, merely 15% of office urologists engaged in performing UDS. Predominant reasons for not performing UDS were limited personnel capacity and inadequate remuneration.

The primary objective of this study was to evaluate reasons for the decline in UDS numbers. The results demonstrated exhausted capacities and prolonged waiting times as main causes in numerous urological departments. Consequently, it is evident that the decline in UDS numbers cannot be attributed to a scarcity of indications, as suggested by studies questioning the necessity of certain UDS indications [10, 11, 13]. The main reason for exhausted capacities is due to shortage of personnel in urological departments and office. This issue is intensified by the widespread shortage of nurses and physicians within the German healthcare system [17]. Consequently, patients with bladder dysfunction often find themselves competing with patients with more urgent medical conditions, such as cancer. Another issue are costs and renumeration aspects, which pose a greater challenge for office urologists compared to clinics (Fig. 1C & D). While initial investments in UDS devices may be more feasible for hospitals, data suggests that smaller hospitals encounter greater hurdles in this regard (Table 2). Nevertheless, major investment costs and poor renumeration remain a relevant problem for office urologists. In 2024, urologists in private practice receive 102.03 € per UDS, which does not adequately cover personnel, time and material expenses [18]. This disparity in costs is evident in the lower utilization of UDS in office urologists compared to urological departments (15% vs. 55%), with a considerable portion of UDS in clinics being provided for referring physicians.

Besides the lack of medical staff, the study highlights another important issue in the health care system: centralisation. The analysis reveals that urological departments with a lower volume of UDS more frequently report a reduction of UDS capacities in the last 5 years, and now shorter waiting times for UDS and underutilization of these capacities compared to departments with high numbers of UDS. This trend indicates a pattern of increasing UDS numbers in larger departments and a reduction in smaller ones, which is concordant with our previous study [9]. Consequently, there has been a trend toward greater centralization, resulting in fewer clinics with high UDS utilization. This leads to the question of whether this trend should be viewed positively. On the one hand, numerous studies show that centralization leads to more routine and expertise, especially in oncological and surgical fields [19, 20]. Given the demanding nature of UDS in terms of execution and interpretation [1, 2, 4], high volume departments are likely to provide more reliable examinations. On the other hand, this centralization means that a considerable number of hospitals (in 2019: 45% of all urological departments) cannot offer UDS in their specialist training programs, leading to lower expertise among future urologists. This loss of importance in training is already reflected in a partially reduction in the number of urodynamics required for specialist training in Germany [21]. This could lead to a more empiric management of functional disorders of the lower urinary tract and potentially delay adequate diagnoses. Functional urology and UDS should be more strongly implemented in training concepts. This often falls by the wayside, as residents focus on the surgical aspects of urology. Nevertheless, it plays a crucial role in the differentiated diagnosis of surgical and conservative therapies [22].

Our study has several limitations. We only analysed UDS in urological departments, not taking gynaecological and paediatric departments as well as rehabilitation centres into account. However, according to our previous study, UDS in gynaecology accounts for only 4% of all UDS. Furthermore, no changes were recorded in the numbers of UDS conducted in children. Therefore, the results in this study are not relevant for paediatric patient. Moreover, rehabilitation centres revealed no change in the numbers of UDS, reflecting the importance of UDS in neuro-urological disorders [9, 23]. Furthermore, surveying the situation in outpatient practices remains challenging due to the generally poor response rate. To circumvent this issue, we used a representative sample. While the response rate in this context may not match that of clinics, it significantly surpasses typical rates, thereby mitigating potential biases. Nevertheless, this is the first study analyzing the declining utilization of urodynamic studies in German urological departments and private practices. Additionally, this study marks the first instance of data collection on UDS utilization. With a response rate of 80% from urological departments, we present an accurate representation of the UDS care reality. Our findings underscore the widespread issue of exhausted capacities, primarily driven by a lack of personnel. Notably, many UDS procedures in urological department were performed for referring urologists. Especially small departments are scaling back their UDS services, leading to a decrease in the number of urological departments offering UDS. Ultimately, addressing this challenge requires careful consideration. While increasing reimbursement for UDS may seem like a straightforward solution, the more pressing issue lies in the shortage of personnel.

Despite the consequences for patients with prolonged waiting times, another important aspect is the relevance of specialist training for future urologists. Urological societies should ensure that the importance of UDS in specialist training is not lost in view of the decreasing number of clinics that are still able to offer this diagnostic procedure.

Further studies should explore the impact on patients resulting from delayed therapies caused by long waiting times for UDS, as well as the effects on urological resident training and expertise in UDS. Given that international studies also indicate a decline in UDS utilization, it would be valuable to investigate whether similar challenges exist in other countries. This could provide insights into potential systemic issues and deliver strategies for addressing them globally [5, 6, 8].

## Conclusion

The declining numbers of UDS and participating urological departments, along with reduced utilization in private practices, stem from constraints in both personnel and finances. This situation can lead to prolonged waiting periods and delayed therapy for patients. Additionally, it may also translate into diminished opportunities for UDS education and hands-on experience during urological specialist training.

## Electronic supplementary material

Below is the link to the electronic supplementary material.

Supplementary Material 1

Supplementary Material 2
