# Supplementary material for: Declining utilization of urodynamic studies in urological care in Germany: time to say goodbye?
Source: World J Urol. 2024 Jul 24;42(1):440. doi: 10.1007/s00345-024-05154-3 (PMC11269447; doi:10.1007/s00345-024-05154-3)
Supplement: Supplementary file 1 — Supplementary Material 1 [file 345_2024_5154_MOESM1_ESM.docx]

**Die Entwicklung der Urodynamischen Untersuchung in deutschen urologischen Kliniken**

Als urodynamische Untersuchungen sind in diesem Fragebogen alle Untersuchungen gemeint, die zumindest das transurethrale Legen einer Messsonde voraussetzen – also keine alleinige Uroflowmetrie.

1. Geben Sie den Versorgungsgrad Ihres Krankenhauses an:
   - Universitätsklinik
   - Spezialisiertes Fachkrankenhaus
   - Klinik für Rehabilitationsmedizin
   - Krankenhaus der Maximalversorgung (≥700 Betten)
   - Krankenhaus der Zentralversorgung (500 – 699 Betten)
   - Krankenhaus der Regelversorgung (300 – 499 Betten)
   - Krankenhaus der Grundversorgung (200 – 299 Betten)
   - Krankenhaus der Mindestversorgung (150 – 199 Betten)
   - Spezialisiertes Fachkrankenhaus
2. Wie viele Betten hat Ihre Fachabteilung?
   - 1 – 10 Betten
   - 11 – 30 Betten
   - 31 – 50 Betten
   - ≥ 51 Betten
3. Ist Ihre Fachabteilung Teil eines zertifizierten Kontinenz- und Beckenbodenzentrums?
   - Ja
   - Nein
4. Wer führt bei Ihnen die urodynamischen Untersuchungen durch (Mehrfachauswahl)?
   - Ärzt/innen
   - Pfleger/innen
   - Urotherapeut/innen
5. Wer wertet bei Ihnen die urodynamischen Untersuchungen aus?
   - Jede Ärztin/Arzt
   - Spezialist/in (1-2 erfahrene Ärzt/innen)
   - Interdisziplinäre Auswertung
6. Geben Sie die Zahl der Urodynamikplätze an, die in Ihrer Fachabteilung zur Verfügung stehen.

Zahl der Urodynamikplätze: ______

1. Welche urodynamischen Untersuchungen führen Sie in Ihrer Fachabteilung durch (Mehrfachauswahl)?
   - Uroflow
   - Urethradruckprofil
   - Zystomanometrie
   - Videourodynamik
2. Wie viele urodynamische Untersuchungen führt Ihre Fachabteilung pro Jahr durch?
   - 1 – 50
   - 51 – 100
   - 101 – 250
   - 251 – 500
   - >500
3. Wie hat sich die Zahl der urodynamischen Untersuchungen in den letzten 5 Jahren in Ihrer Fachabteilung entwickelt? Die Zahl der urodynamischen Untersuchungen…
   - … hat abgenommen.
   - … ist gleichgeblieben.
   - … hat zugenommen.
4. Wie lange beträgt die durchschnittliche Wartezeit für Termine zu urodynamischen Untersuchungen für Patient/innen in Ihrer Fachabteilung?
   - < 1 Woche
   - 1 – 4 Wochen
   - 1 – 3 Monate
   - 4 – 6 Monate
   - > 6 Monate
5. Wie hoch ist der Anteil an urodynamischen Untersuchungen, die Sie als Auftragsleistung für externe Zuweiser durchführen?
   - Wir führen keine Untersuchungen für externe Zuweiser durch
   - 1 – 25%
   - 26 – 50%
   - 51 – 75%
   - 75 – 100%
6. Sind Ihre Kapazitäten für urodynamische Untersuchungen in der Regel ausgeschöpft?
   - Ja
   - Nein (weiter ab Frage 14)
7. Wie sehr schränken folgende Faktoren die Kapazität der Urodynamiken in Ihrer Fachabteilung ein?

|  | Gar nicht | gering | mittel | hoch |
| --- | --- | --- | --- | --- |
| Aufwand an Pflegepersonal | 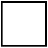 | 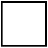 | 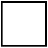 | 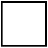 |
| Aufwand an ärztlichen Personal | 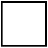 | 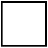 | 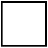 | 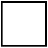 |
| Struktureller Aufwand (Räumlichkeiten, Anzahl der Geräte) | 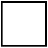 | 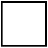 | 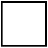 | 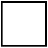 |
| Untersuchungskosten | 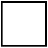 | 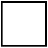 | 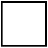 | 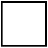 |
| Vergütung | 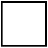 | 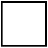 | 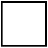 | 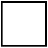 |

1. Planen Sie in Ihrer Fachabteilung eine Änderung der Kapazität an urodynamischen Untersuchungen in den nächsten 3 Jahren?
   - Ja, die Zahl der urodynamischen Untersuchungen soll reduziert werden
   - Ja, die Zahl der urodynamischen Untersuchungen soll gesteigert werden
   - Nein
2. Geben Sie den Anteil von Erkrankungsbildern an, bei denen Sie urodynamische Untersuchungen durchführen bzw. dazu überweisen (Angaben in Prozent – es muss insgesamt 100% ergeben):

Unkomplizierte Belastungsinkontinenz der Frau ____ %

Komplizierte Belastungsinkontinenz der Frau ____ %

Rezidivinkontinenz ____ %

Deszensus der Frau ____ %

Überaktive Harnblase ____ %

Neurogene Blasenfunktionsstörungen ____ %

Postoperative Blasenfunktionsstörungen ____ %

1. In den letzten 10 Jahren gab es Studien die die Indikation der Urodynamik bei Frauen vor einer Belastungsinkontinenzoperation untersucht haben. Bei welchen Patientinnen führen Sie urodynamische Untersuchungen durch?
   - Alle Frauen vor einer Operation der Belastungsinkontinenz
   - Nur Frauen vor einer Operation der Belastungsinkontinenz mit Auffälligkeiten in der Basisdiagnostik
