# Supplementary material for: Declining utilization of urodynamic studies in urological care in Germany: time to say goodbye?
Source: World J Urol. 2024 Jul 24;42(1):440. doi: 10.1007/s00345-024-05154-3 (PMC11269447; doi:10.1007/s00345-024-05154-3)
Supplement: Supplementary file 2 — Supplementary Material 2 [file 345_2024_5154_MOESM2_ESM.docx]

**Die Entwicklung von urodynamischen Untersuchungen in deutschen urologischen Praxen**

1. Geben Sie Ihre Praxisgröße an
   - Einzelpraxis
   - Gemeinschaftspraxis
   - MVZ
2. Wie viele Patienten betreuen Sie pro Quartal?
   - < 500
   - 500 – 1000
   - 1000 – 2000
   - > 2000
3. Geben Sie den Anteil von Erkrankungsbildern an, bei denen Sie urodynamische Untersuchungen durchführen bzw. dazu überweisen

(Angaben in Prozent – es muss insgesamt 100% ergeben):

Belastungsinkontinenz der Frau ____ %

Belastungsinkontinenz des Mannes ____ %

LUTS des Mannes ____ %

Überaktive Harnblase ____ %

Neurogene Blasenentleerungsstörungen ____ %

1. In den letzten 10 Jahren gab es Studien die die Indikation der Urodynamik bei Frauen vor einer Belastungsinkontinenzoperation und bei Männern vor einer Operation bei obstruktiven Beschwerden untersucht haben. Bei welchen Patient/innen führen Sie urodynamische Untersuchungen durch?
   - Alle Frauen vor einer Operation der Belastungsinkontinenz
   - Nur Frauen vor einer Operation der Belastungsinkontinenz mit Auffälligkeiten in der Basisdiagnostik
   - Alle Männer vor einer Operation bei obstruktiven Beschwerden
   - Nur Männer vor einer Operation bei obstruktiven Beschwerden mit Auffälligkeiten in der Basisdiagnostik
2. Haben Sie einen Urodynamikplatz in ihrer Praxis?
   - Ja, und ich nutze ihn (weiter ab Frage 10)
   - Ja, aber ich nutze ihn nicht mehr (weiter ab Frage 6)
   - Nein, ich habe keinen Urodynamikplatz (weiter ab Frage 6)

Bitte umblättern ⇒

**Antwortblock für Praxen ohne Urodynamiken**

1. Welche Faktoren halten sie davon ab, Urodynamiken in Ihrer Praxis durchzuführen?

|  | Gar nicht | gering | mittel | hoch |
| --- | --- | --- | --- | --- |
| Aufwand an Personal | 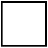 | 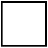 | 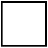 | 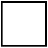 |
| Struktureller Aufwand  (Räumlichkeiten, Anzahl der Geräte) | 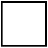 | 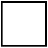 | 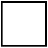 | 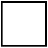 |
| Anschaffungskosten | 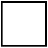 | 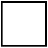 | 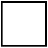 | 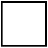 |
| Vergütung | 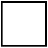 | 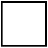 | 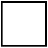 | 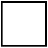 |
| Fachliche Auseinandersetzung mit dem Thema | 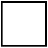 | 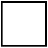 | 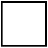 | 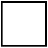 |

1. Wohin schicken Sie Patienten zur Durchführung einer Urodynamikuntersuchung?
   - Eine andere Praxis
   - In eine urologische Klinik
   - In eine gynäkologische Klinik
   - In ein Kontinenz- und Beckenbodenzentrum
2. Wie viele Überweisungen zur Urodynamik stellen Sie jährlich ca. aus?

Freie Antwort: _____

1. Wie lange ist die Wartezeit für Urodynamiken beim Leistungserbringer?

Freie Antwort: _____

Bitte weiter ab Frage 19

**Antwortblock für Praxen mit Urodynamiken**

1. Geben Sie die Zahl der Urodynamikplätze in Ihrer Praxis an?

Freie Antwort: _____

1. Wer führt bei Ihnen die urodynamischen Untersuchungen durch?
   - Ärzt/innen
   - Arzthelfer/innen
   - Urotherapeut/innen
   - Mitarbeiter/in eines Urodynamikgeräteanbieters
2. Welche urodynamischen Untersuchungen führen Sie in Ihrer Praxis durch (Mehrfachauswahl):
   - Uroflow
   - Urethradruckprofil
   - Zystomanometrie
   - Flow-EMG
   - Druck-Fluss-Messung
   - Provokationstests

(z.B. Eiswassertests)

- - Videourodynamik

1. Wie viele urodynamischen Untersuchungen führt Ihre Praxis pro Jahr durch?
   - 1 – 25
   - 25 – 50
   - 51 – 100
   - > 100
2. Wie hat sich die Zahl der urodynamischen Untersuchungen in den letzten 5 Jahren in Ihrer Praxis entwickelt? Die Zahl der urodynamischen Untersuchungen…
   - … hat abgenommen.
   - … ist gleichgeblieben.
   - … hat zugenommen.
3. Wie lange beträgt die durchschnittliche Wartezeit für Termine zu urodynamischen Untersuchungen für Patient/innen in Ihrer Praxis?

Freie Antwort: _____

1. Sind Ihre Kapazitäten für urodynamische Untersuchungen in der Regel ausgeschöpft?
   - Ja (weiter Frage 18 - 20)
   - Nein

1. Wie sehr schränken folgende Faktoren die Kapazität der Urodynamiken in Ihrer Praxis ein?

|  | Gar nicht | gering | mittel | hoch |
| --- | --- | --- | --- | --- |
| Aufwand an Personal | 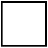 | 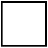 | 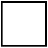 | 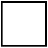 |
| Struktureller Aufwand  (Räumlichkeiten, Anzahl der Geräte) | 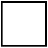 | 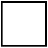 | 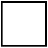 | 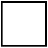 |
| Untersuchungskosten | 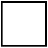 | 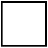 | 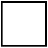 | 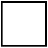 |
| Vergütung | 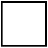 | 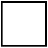 | 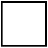 | 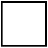 |
| Hygieneanforderungen | 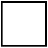 | 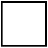 | 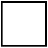 | 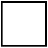 |

1. Planen Sie in Ihrer Praxis/MVZ eine Änderung der Kapazität an urodynamischen Untersuchungen in den nächsten 3 Jahren?
   - Ja, die Zahl der urodynamischen Untersuchungen soll reduziert werden
   - Ja, die Zahl der urodynamischen Untersuchungen soll gesteigert werden
   - Nein

Bitte umblättern ⇒

**Abschließende Fragen:**

1. Würden Sie mehr urodynamische Untersuchungen durchführen (lassen), wenn Sie oder die Leistungserbringer mehr Kapazitäten hätten?
   - Ja
   - Nein
2. Gibt es Aspekte zum Thema Urodynamik, die Sie uns noch mitteilen möchten?

(Freie Antworten):

____________________________________________________________________________

____________________________________________________________________________

____________________________________________________________________________

____________________________________________________________________________

____________________________________________________________________________

____________________________________________________________________________

____________________________________________________________________________

____________________________________________________________________________

____________________________________________________________________________

____________________________________________________________________________

____________________________________________________________________________

____________________________________________________________________________
